# Measuring treatment effect on psoriatic arthritis-related domains: insights from the SPIRIT-H2H study at weeks 24 and 52

**DOI:** 10.1007/s10067-021-05891-5

**Published:** 2021-09-13

**Authors:** Frank Behrens, Soyi Liu Leage, Christophe Sapin, Celine El Baou, Inmaculada De La Torre, Gabriella Meszaros, Georg Schett, Bernard Combe, Filip van den Bosch, Laure Gossec

**Affiliations:** 1 Dpt. of Rheumatology, Goethe University and Fraunhofer ITMP and CIMD, 60590 Frankfurt am Main, Germany; 2 Eli Lilly and Company, Indianapolis, USA; 3 Friedrich-Alexander-University Erlangen-Nuremberg and Universitätsklinikum, Erlangen, Germany; 4 Deutsches Zentrum Für Immuntherapie, Friedrich-Alexander-University Erlangen-Nuremberg, Erlangen, Germany; 5 Montpellier University, Montpellier, France; 6 Ghent University Hospital, Ghent, Belgium; 7 Sorbonne Université, INSERM, Institut Pierre Louis D’Epidémiologie Et de Santé Publique, Paris, France; 8 Rheumatology Department, Pitié-Salpêtrière Hospital, AP-HP Sorbonne Université, Paris, France

**Keywords:** Musculoskeletal abnormalities, Psoriasis, Psoriatic arthritis, Quality of life

## Abstract

**Introduction:**

Improvements in both musculoskeletal and non-musculoskeletal manifestations are important treatment goals in psoriatic arthritis (PsA).

**Objective:**

These post hoc analyses determined whether additional benefits related to various PsA domains are observed in patients simultaneously achieving 50% improvement in American College of Rheumatology criteria (ACR50) and 100% improvement in Psoriasis Area Severity Index (PASI100), the primary endpoint of the SPIRIT-H2H study.

**Methods:**

Patients with active PsA and psoriasis in SPIRIT-H2H (*N* = 566) were categorised into two sets of four response groups irrespective of treatment allocation (approved dosages of ixekizumab or adalimumab): patients who simultaneously achieved ACR50 and PASI100 response, achieved ACR50 response only, achieved PASI100 response only, or did not achieve ACR50 or PASI100 response after 24 and 52 weeks of treatment. Patients achieving simultaneous ACR50 and PASI100 response were compared with the other patient response groups at the corresponding time point for efficacy and health-related quality of life (HRQoL) outcomes.

**Results:**

Patients simultaneously achieving ACR50 and PASI100 responses at week 24 or 52 showed higher rates of ACR70 response, minimal disease activity, Disease Activity in Psoriatic Arthritis ≤ 4, resolution of enthesitis and dactylitis, and HRQoL improvement at weeks 24 and 52, respectively, than the other corresponding response groups at both time points.

**Conclusion:**

High levels of disease control, such as those obtained with simultaneous achievement of ACR50 and PASI100 response, were linked to better outcomes across a wide range of endpoints that are important for patients with PsA. Patients meeting this combined endpoint showed more comprehensive and thus greater control of disease activity.

Trial registration

NCT03151551

**Table:** 

**Key Points** • *Treatment goals for patients with psoriatic arthritis emphasise the importance of improving both musculoskeletal and non-musculoskeletal manifestations of the disease*. • *A combined endpoint considering both these manifestations, achievement of at least 50% improvement in American College of Rheumatology criteria and 100% improvement in Psoriasis Area Severity Index, was linked with achievement of a number of other endpoints relevant to psoriatic arthritis, including health-related quality of life that are important to patients with psoriatic arthritis*. • *Patients meeting the combined endpoint were more likely to achieve a disease state of remission, which is the stated aim of treatment for psoriasis*.

**Supplementary Information:**

The online version contains supplementary material available at 10.1007/s10067-021-05891-5.

## Introduction

Psoriatic arthritis (PsA) is a chronic inflammatory disease that presents with both musculoskeletal and non-musculoskeletal manifestations [1, 2]. Musculoskeletal manifestations of PsA include peripheral arthritis, spondylitis, dactylitis, and enthesitis, whereas non-musculoskeletal manifestations include substantial skin and nail disease that can range from mild to severe in intensity. PsA is also associated with inflammatory bowel disease and uveitis. In addition, patients with PsA show impairment in physical, work, and social activities.

The Group for Research and Assessment of Psoriasis and Psoriatic Arthritis (GRAPPA), the European League Against Rheumatism (EULAR), and the American College of Rheumatology (ACR)/National Psoriasis Foundation share common treatment goals for patients with PsA: to improve health-related quality of life (HRQoL); achieve robust improvement of musculoskeletal manifestations; prevent structural damage; and control non-musculoskeletal manifestations, including skin disease [2–4]. Kavanaugh et al. [5] confirmed that improvement in both joint and skin manifestations is necessary to optimise improvement in HRQoL. For this reason, a combination of two validated outcome measures (≥ 50% improvement in ACR criteria [ACR50] and 100% improvement in Psoriasis Area Severity Index score [PASI100]) was defined as the primary endpoint in the phase IIIb/IV 52-week head-to-head SPIRIT-H2H trial (NCT03151551) of the interleukin (IL)-17A antagonist ixekizumab and the tumour necrosis factor (TNF) inhibitor adalimumab [6]. Ixekizumab is a high-affinity monoclonal antibody that selectively targets IL-17A; it is approved in the USA and European Union for the treatment of moderate-to-severe plaque psoriasis (PsO) in adults and children aged ≥ 6 years and PsA and axial spondyloarthritis in adults [7, 8].

SPIRIT-H2H enrolled patients with active PsA and PsO who were naïve to biologic disease-modifying antirheumatic drugs (bDMARDs) and had experienced an inadequate response to conventional synthetic disease-modifying antirheumatic drugs (csDMARDs). The primary and major secondary objectives of the study were achieved at week 24, with ixekizumab being superior to adalimumab for the percentage of patients who simultaneously achieved an ACR50 and PASI100 response, non-inferior for ACR50 response, and superior for PASI100 response [6]. In addition, the efficacy of both treatments was maintained at 52 weeks, with a significantly higher proportion of patients treated with ixekizumab versus adalimumab simultaneously achieving ACR50 and PASI100 at this time (39 versus 26%, *p* < 0.001) [9]. Ixekizumab also showed greater efficacy than adalimumab for improvement in a number of additional musculoskeletal, non-musculoskeletal, and HRQoL outcomes at week 24 and/or week 52 [6, 9].

The primary endpoint of SPIRIT-H2H (simultaneous achievement of an ACR50 and PASI100 response) has not been validated. Therefore, to determine whether additional benefits related to various PsA domains were observed in patients simultaneously achieving ACR50 and PASI100 at weeks 24 and 52, this analysis aimed to describe efficacy and HRQoL outcomes in patients achieving versus not achieving the primary endpoint at weeks 24 and 52 in the overall SPIRIT-H2H study population, irrespective of treatment allocation.

## Materials and methods

### SPIRIT-H2H design

The design of SPIRIT-H2H (NCT03151551) has already been presented [6] and is therefore described only briefly here. SPIRIT-H2H was a 52-week, multicentre, randomised, open-label, assessor-blinded, parallel-group study evaluating the efficacy and safety of ixekizumab versus adalimumab in adult patients fulfilling Classification for Psoriatic Arthritis criteria for active PsA (tender joint count ≥ 3/68, swollen joint count ≥ 3/66) and with active PsO (body surface area [BSA] involvement of ≥ 3%) who had an inadequate response to ≥ 1 csDMARDs and were naïve to bDMARDs [6]. Patients were randomised (1:1 and stratified by the presence of moderate-to-severe PsO and concomitant csDMARD use) to the approved dosage regimens of ixekizumab or adalimumab based on the presence/absence of moderate-to-severe PsO [6]. Moderate-to-severe PsO was defined as PASI ≥ 12 with a static Physician Global Assessment ≥ 3 and BSA involvement of ≥ 10% at baseline. A stable dose of csDMARDs, including methotrexate, could be maintained throughout the study if the patient was receiving such treatment at screening.

After the week-24 database lock and initial analysis run, nine patients with active PsO and BSA ≥ 3% were found to have been assessed as PASI = 0 at baseline, a medical inconsistency that was resolved using medical judgement. These patients were considered PASI100 responders if PASI = 0 and BSA = 0 at postbaseline visits.

SPIRIT-H2H was conducted in accordance with the ethical principles of the Declaration of Helsinki; all patients provided written informed consent and the study protocol was approved by the ethical review board (17/LO/0794) prior to the start of study-related procedures.

### Current analyses

The current post hoc analyses report efficacy and HRQoL endpoints in four independent analysis groups based on achievement of study endpoints, including a simultaneous ACR50 and PASI100 response (the primary endpoint), using the overall SPIRIT-H2H intention-to-treat (ITT) population, irrespective of treatment group. The four response groups were combined responder (CR24; patients who achieved simultaneous ACR50 and PASI100 response), joint responder (JR24; patients who achieved ACR50 but not PASI100 response), skin responder (SR24; patients who achieved PASI100 but not ACR50 response), and non-responder (NR24; patients who did not achieve ACR50 or PASI100 response after 24 weeks of treatment). Patients were also reallocated into the four groups based on responses after 52 weeks of treatment (CR52, JR52, SR52, and NR52, respectively). Patients who withdrew before week 24 or 52 were allocated to the groups who did not achieve an ACR50 or PASI100 response, based on non-responder imputation principles.

### Analysis endpoints

Musculoskeletal endpoints were the proportions of patients at weeks 24 and 52 who achieved a simultaneous ACR50 and PASI100 response; an ACR50, ACR20, and ACR70 response; Disease Activity in Psoriatic Arthritis (DAPSA) ≤ 4 (remission); DAPSA ≤ 14 (low disease activity or remission); minimal disease activity (MDA; 5/7 criteria) and 7/7 MDA criteria (very low disease activity [VLDA]); resolution of enthesitis (Spondyloarthritis Research Consortium of Canada [SPARCC] Enthesitis Index = 0 or Leeds Enthesitis Index [LEI] = 0) among patients with enthesitis at baseline (SPARCC Enthesitis Index > 0 or LEI > 0); resolution of dactylitis (Leeds Dactylitis Index-Basic [LDI-B] = 0) among patients with dactylitis at baseline (LDI-B > 0); and no impairment of physical function (Health Assessment Questionnaire-Disability Index [HAQ-DI] ≤ 0.5). Additionally, change from baseline was evaluated for SPARCC and LEI among patients with enthesitis at baseline, LDI-B among patients with dactylitis at baseline, HAQ-DI, and 36-Item Short Form Survey (SF-36) physical component score (PCS) and SF-36 mental component score (MCS).

Non-musculoskeletal endpoints were the proportions of patients at weeks 24 and 52 who achieved a PASI75, PASI90, and PASI100 response; resolution of fingernail psoriasis (Nail Psoriasis Severity Index [NAPSI] fingernails = 0) among patients with fingernail involvement at baseline (NAPSI > 0); and a Dermatology Life Quality Index score of 0 or 1 (DLQI 0,1).

### Analysis methods

Baseline characteristics of each response group were summarised and compared descriptively (mean and standard deviation for categorical outcomes, and absolute and relative frequencies for categorical outcomes) for the week-24 and week-52 groups.

Categorical endpoints were examined at week 24 according to response group at week 24. The proportions of patients achieving responses to categorical endpoints were analyzed using logistic regression with non-responder imputation in the ITT population and included treatment, concomitant csDMARD use at baseline, and moderate-to-severe PsO involvement as factors. Differences between CR24 (who simultaneously achieved an ACR50 and PASI100 response at week 24) and each of the other three response groups at week 24 (JR24, SR24, and NR24) were also evaluated using Fisher’s exact test. Similar analyses were performed at week 52 for CR52 (who simultaneously achieved an ACR50 and PASI100 response at week 52) compared with each of the other three response groups at week 52 (JR52, SR52, and NR52).

Adjusted change from baseline in endpoints of interest in each response group was evaluated using an analysis of covariance model that included treatment, moderate-to-severe PsO at baseline, and baseline value as factors. Missing data were imputed using a modified baseline observation carried forward approach. Differences in least squares mean change were evaluated using *t*-tests.

Analyses were performed using SAS Enterprise Guide 7.15. Sankey diagrams were created to visualise the trajectory of response at week 52.

## Results

### Patient characteristics

A total of 566 bDMARD-naïve patients with active PsA and PsO were included in these analyses; 283 patients received ixekizumab and 283 patients received adalimumab. When categorised by response at week 24, 181 patients simultaneously achieved an ACR50 and PASI100 response (CR24), 94 patients achieved an ACR50 but not a PASI100 response (JR24), 121 patients achieved a PASI100 response but not an ACR50 response (SR24), and 170 patients did not achieve an ACR50 or PASI100 response (NR24). When grouped according to response at 52 weeks, 185 patients simultaneously achieved an ACR50 and PASI100 response (CR52), 97 patients achieved an ACR50 but not a PASI100 response (JR52), 114 patients achieved a PASI100 response but not an ACR50 response (SR52), and 170 patients did not achieve an ACR50 or PASI100 response (NR52).

**Fig. 1:**
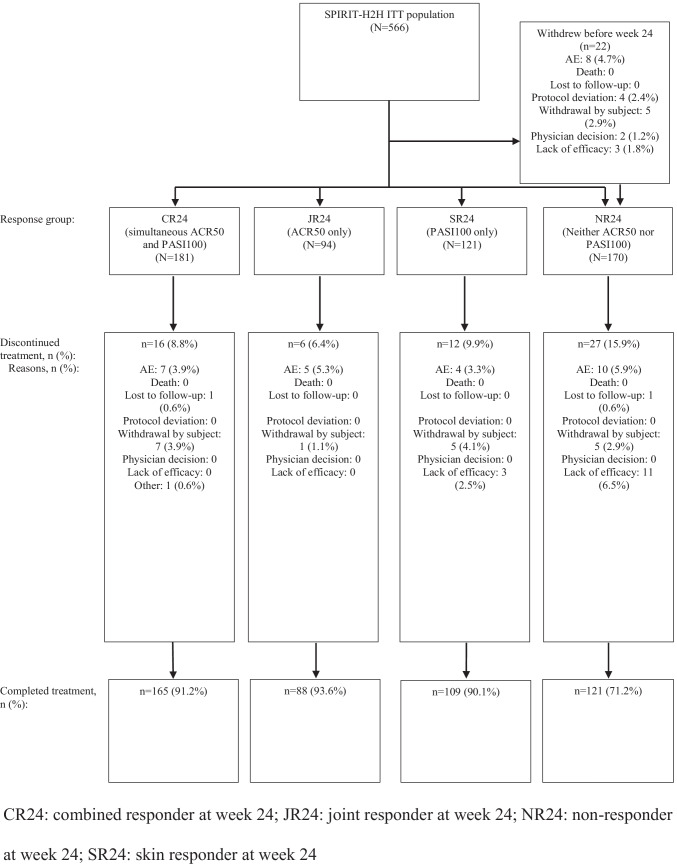
Patient disposition from week 24 to week 52

A total of 483 patients (85.3%) completed the 52-week treatment period (90.1–93.6% of patients in CR24, JR24, and SR24 [who had any combination of ACR50 or PASI100 response at week 24]; Fig. 1). Overall, 22 of the 566 patients in the ITT population (3.9%) discontinued treatment before week 24, and 61 patients (10.8%) discontinued treatment between weeks 24 and 52.

Numerical differences were noted in baseline characteristics between the week-24 response groups, although few clear trends were observed (Table 1). JR24 had the highest scores of all the response groups for many baseline musculoskeletal-related variables, such as tender and swollen joint counts, and DAPSA, enthesitis, dactylitis, HAQ-DI and modified Composite Psoriatic Disease Activity Index scores. This group also had the highest scores for some baseline non-musculoskeletal-related variables, such as PASI score, BSA-affected, and DLQI score, and had the greatest proportion of patients with nail involvement (NAPSI score > 0). Use of csDMARDs and methotrexate was highest, and the presence of enthesitis was lowest, in CR24. The proportion of patients with C-reactive protein level > 6 mg/L was lowest in SR24. At baseline, NR24 had a longer mean duration of symptoms since diagnosis for both PsO and PsA, the highest body mass index and the lowest use of csDMARDs.

**Table 1:** Baseline characteristics of week-24 response groups^a^

	CR24 (ACR50 plus PASI100) (*N* = 181)	JR24 (ACR50 only) (*N* = 94)	SR24 (PASI100 only) (*N* = 121)	NR24 (neither ACR50 nor PASI100) (*N* = 170)
Age (years)	46.3 ± 11.5	48.9 ± 12.6	48.4 ± 12.8	48.6 ± 12.1
Sex, male	110 (60.8)	61 (64.9)	52 (43.0)	89 (52.4)
BMI (kg/m^2^)	28.8 ± 6.7	29.3 ± 5.6	29.5 ± 7.2	31.5 ± 9.4
Duration of symptoms (years)				
Since Ps diagnosis	14.2 ± 11.2	14.9 ± 12.3	14.3 ± 13.1	17.6 ± 14.3
Since PsA diagnosis	5.8 ± 6.6	5.7 ± 6.1	6.2 ± 6.9	7.0 ± 7.6
csDMARD use	137 (75.7)	63 (67.0)	86 (71.1)	106 (62.4)
Methotrexate use	119 (65.7)	52 (55.3)	73 (60.3)	92 (54.1)
Glucocorticoid use	39 (21.5)	15 (16.0)	19 (15.7)	38 (22.4)
Tender joint count	18.8 ± 13.0	22.4 ± 14.8	20.2 ± 14.1	20.4 ± 14.8
Swollen joint count	11.0 ± 8.6	12.0 ± 8.6	9.7 ± 5.9	9.4 ± 7.5
Physician’s disease activity VAS (mm)	59.8 ± 18.7	61.8 ± 17.0	56.8 ± 18.2	58.6 ± 17.0
Patient’s disease activity VAS (mm)	62.7 ± 18.8	68.6 ± 18.7	60.8 ± 22.8	64.4 ± 21.4
Patient’s pain VAS (mm)	60.5 ± 21.2	66.0 ± 18.0	58.0 ± 23.5	60.9 ± 22.0
HAQ-DI score	1.2 ± 0.6	1.4 ± 0.6	1.2 ± 0.7	1.2 ± 0.8
CRP level (mg/L)	11.9 ± 21.0	11.8 ± 18.0	7.1 ± 10.8	9.7 ± 13.6
CRP level > 6 mg/L	76 (42.2)	39 (43.3)	34 (29.1)	69 (41.8)
DAPSA score	43.4 ± 21.4	49.3 ± 23.8	42.2 ± 19.8	43.8 ± 23.2
DAS28-CRP score	4.8 ± 1.1	5.1 ± 1.0	4.7 ± 1.0	4.8 ± 1.1
LEI > 0	87 (48.1)	54 (57.4)	71 (58.7)	94 (55.6)
LEI score^b^	2.5 ± 1.5	2.8 ± 1.5	2.6 ± 1.5	2.7 ± 1.3
SPARCC > 0	98 (54.1)	64 (68.1)	86 (71.1)	112 (66.3)
SPARCC score^b^	4.8 ± 3.6	6.0 ± 3.6	5.3 ± 3.7	5.3 ± 3.6
LDI-B > 0	34 (18.8)	20 (21.3)	21 (17.4)	25 (14.8)
LDI-B score^b^	50.8 ± 83.2	84.2 ± 193.5	41.1 ± 40.1	25.7 ± 17.2
mCPDAI score	6.0 ± 2.1	6.6 ± 2.1	6.1 ± 1.9	6.1 ± 2.0
PASI score	7.6 ± 9.0	10.1 ± 9.1	6.1 ± 5.8	7.9 ± 7.5
sPGA score	2.6 ± 0.9	2.9 ± 0.8	2.6 ± 0.9	2.7 ± 0.8
BSA % affected	14.2 ± 18.8	16.6 ± 17.8	10.2 ± 11.0	14.5 ± 17.9
NAPSI > 0	113 (62.4)	70 (74.5)	83 (68.6)	102 (60.4)
NAPSI score^b^	20.0 ± 16.4	19.1 ± 17.7	18.0 ± 16.8	20.0 ± 18.9
DLQI score	9.3 ± 7.8	12.3 ± 7.7	8.7 ± 7.1	9.7 ± 7.4
SF-36 PCS	37.7 ± 8.1	35.1 ± 7.2	37.7 ± 8.8	36.8 ± 9.3
SF-36 MCS	45.0 ± 11.1	43.2 ± 10.8	44.2 ± 10.7	44.4 ± 12.3

Results are expressed as mean ± SD or *n* (%); percentages are calculated based on numbers of patients providing data

^a^The four response groups were combined responder (CR24; patients who achieved simultaneous ACR50 and PASI100 response), joint responder (JR24; patients who achieved ACR50 but not PASI100 response), skin responder (SR24; patients who achieved PASI100 but not ACR50 response), and non-responder (NR24; patients who did not achieve ACR50 or PASI100 response after 24 weeks of treatment)

^b^Mean ± SD score in patients with baseline score > 0 (see row above for numbers of patients evaluated)

*ACR50*, ≥ 50% improvement in American College of Rheumatology criteria; *BMI*, body mass index; *BSA*, body surface area; *CR24*, combined responder at week 24; *CRP*, C-reactive protein; *csDMARD*, conventional synthetic disease-modifying antirheumatic drug; *DAPSA*, Disease Activity for Psoriatic Arthritis; *DAS28-CRP*, Disease Activity Score with CRP; *DLQI*, Dermatology Life Quality Index; *HAQ-DI*, Health Assessment Questionnaire-Disability Index; *JR24*, joint responder at week 24; *LDI-B*, Leeds Dactylitis Index-Basic; *LEI*, Leeds Enthesitis Index; *mCPDAI*, modified Composite Psoriatic Disease Activity Index (without spinal disease assessment); *MCS*, mental component score; *NAPSI*, Nail Psoriasis Severity Index; *NR24*, non-responder at week 24; *PASI*, Psoriasis Area Severity Index; *PASI100*, 100% improvement in PASI; *PCS*, physical component score; *Ps*, psoriasis: *PsA*, psoriatic arthritis; *SD*, standard deviation; *SF-36*, 36-Item Short Form Survey; *SPARCC*, Spondyloarthritis Research Consortium of Canada; *sPGA*, static Physician’s Global Assessment of Psoriasis; *SR24*, skin responder at week 24; *VAS*, visual analogue scale

Baseline characteristic findings were similar when patients were grouped according to response at 52 weeks (Table S1).

### Clinical and patient-reported outcomes according to response at weeks 24 and 52

#### Musculoskeletal outcomes in patients simultaneously achieving an ACR50 and PASI100 response (CR24 and CR52)

Patients in CR24 or CR52, by definition, achieved both ACR20 and ACR50 responses at weeks 24 and 52, respectively. Notably, 64.6% of those in CR24 also achieved an ACR70 response at week 24; 74.6% of those in CR52 also achieved an ACR70 response at week 52 (Table 2).

**Table 2:** Efficacy and health-related quality-of-life outcomes in each response group, irrespective of treatment

Week-24 response group					Week-52 response group			
	CR24 (ACR50 plus PASI100) (*N* = 181)	JR24 (ACR50 only) (*N* = 94)	SR24 (PASI100 only) (*N* = 121)	NR24 (neither ACR50 nor PASI100) (*N* = 170)	CR52 (ACR50 plus PASI100) (*N* = 185)	JR52 (ACR50 only) (*N* = 97)	SR52 (PASI100 only) (*N* = 114)	NR52 (neither ACR50 nor PASI100) (*N* = 170)
Percentage of patients achieving at week 24					Percentage of patients achieving at week 52			
ACR50/PASI100	100	0**	0**	0**	100	0**	0**	0**
ACR20	100	100	53.7**	34.7**	100	100	54.4**	28.2**
ACR50	100	100	0**	0**	100	100	0**	0**
ACR70	64.6	48.9*	0**	0**	74.6	60.8*	0**	0**
DAPSA ≤ 14	92.3	81.9*	43.0**	28.8**	94.6	89.7	39.5**	19.4**
LEI = 0^a^	*n* = 87	*n* = 54	*n* = 71	*n* = 94	*n* = 90	*n* = 56	*n* = 68	*n* = 92
	77.0	75.9	45.1**	38.3**	84.4	83.9	47.1**	29.3**
LDI-B = 0^a^	*n* = 34	*n* = 20	*n* = 21	*n* = 25	*n* = 34	*n* = 19	*n* = 16	*n* = 31
	100	95.0	95.2	72.0**	100	100	81.3*	51.6**
HAQ-DI ≤ 0.5	75.7	64.9	29.8**	25.3**	78.4	61.9*	34.2**	14.7**
HAQ-DI MCID improvement ≥ 0.35	82.3	84.0	41.3**	32.9**	83.2	81.4	48.2**	25.9**
PASI75	100	60.6**	100	37.1**	100	70.1**	100	28.8**
PASI90	100	36.2**	100	14.7**	100	34.0**	100	15.9**
PASI100	100	0**	100	0**	100	0**	100	0**
NAPSI = 0^a^	*n* = 113	*n* = 70	*n* = 83	*n* = 102	*n* = 121	*n* = 68	*n* = 71	*n* = 108
	62.8	50.0	59.0	43.1*	80.2	67.6	73.2	35.2**
DLQI 0,1	77.3	47.9**	70.2	30.0**	82.2	47.4**	64.0**	20.0**
	LS mean improvement (SE) from baseline to week 24 in the following scores				LS mean improvement (SE) from baseline to week 52 in the following scores			
LEI^a^	*n* = 87	*n* = 54	*n* = 71	*n* = 94	*n* = 90	*n* = 56	*n* = 68	*n* = 92
	2.1 (0.2)	2.3 (0.2)	1.4 (0.2)**	1.3 (0.1)**	2.4 (0.1)	2.3 (0.2)	1.4 (0.1)**	1.5 (0.1)**
SPARCC^a^	*n* = 98	*n* = 64	*n* = 86	*n* = 112	*n* = 100	*n* = 68	*n* = 79	*n* = 113
	4.1 (0.3)	4.9 (0.4)	2.5 (0.3)**	2.4 (0.3)**	4.7 (0.3)	4.9 (0.3)	2.6 (0.3)**	2.8 (0.2)**
LDI-B^a^	*n* = 34	*n* = 20	*n* = 21	*n* = 25	*n* = 34	*n* = 19	*n* = 16	*n* = 31
	49.2 (1.1)	48.1 (1.3)	48.5 (1.3)	45.0 (1.2)*	49.1 (2.4)	49.4 (3.2)	49.2 (3.5)	37.2 (2.5)**
HAQ-DI^b^	*n* = 164	*n* = 90	*n* = 104	*n* = 148	*n* = 166	*n* = 92	*n* = 99	*n* = 149
	0.98 (0.04)	0.93 (0.05)	0.43 (0.05)**	0.28 (0.04)**	1.00 (0.04)	0.87 (0.05)*	0.44 (0.05)**	0.29 (0.04)**
NAPSI^a^	*n* = 113	*n* = 70	*n* = 83	*n* = 102	*n* = 121	*n* = 68	*n* = 71	*n* = 108
	16.5 (1.1)	13.0 (1.3)*	15.2 (1.3)	10.7 (1.1)**	17.5 (0.9)	15.7 (1.2)	17.4 (1.2)	9.5 (1.0)**
SF-36	*n* = 180	*n* = 94	*n* = 118	*n* = 165	*n* = 185	*n* = 97	*n* = 114	*n* = 165
SF-36 PCS	13.0 (0.6)	12.7 (0.8)	6.2 (0.7)**	4.6 (0.6)**	13.0 (0.5)	11.8 (0.7)	6.0 (0.7)**	4.0 (0.5)**
SF-36 MCS	5.9 (0.7)	6.4 (0.9)	2.3 (0.9)**	2.1 (0.7)**	7.9 (0.7)	7.2 (0.9)	2.4 (0.9)**	1.0 (0.7)**

^a^Percentage of patients achieving outcome/mean ± SD improvement in score in patients with baseline score > 0

^b^Mean ± SD improvement in score in patients with baseline HAQ-DI ≥ 0.35

*ACR20/50/70*, improvement of ≥ 20%/50%/70% in American College of Rheumatology criteria; *CR24/52*, combined responder at week 24/52; *DAPSA*, Disease Activity for Psoriatic Arthritis; *DLQI*, Dermatology Life Quality Index; *HAQ-DI*, Health Assessment Questionnaire-Disability Index; *JR24/52*, joint responder at week 24/52; *LDI-B*, Leeds Dactylitis Index-Basic; *LEI*, Leeds Enthesitis Index; *LS*, least squares; *MCID*, minimal clinically important difference; *MCS*, mental component score; *NAPSI*, Nail Psoriasis Severity Index; *NR24/52*, non-responder at week 24/52; *PASI75/90/100*, ≥ 75%/90%/100% improvement in Psoriasis Area Severity Index score; *PCS*, physical component score; *SD*, standard deviation; *SE*, standard error; *SF-36*, 36-Item Short Form Survey; *SPARCC*, Spondyloarthritis Research Consortium of Canada; *SR24/52*, skin responder at week 24/52; *p < 0.05, **p ≤ 0.001 versus ACR50 plus PASI100 response group

**Fig. 2:**
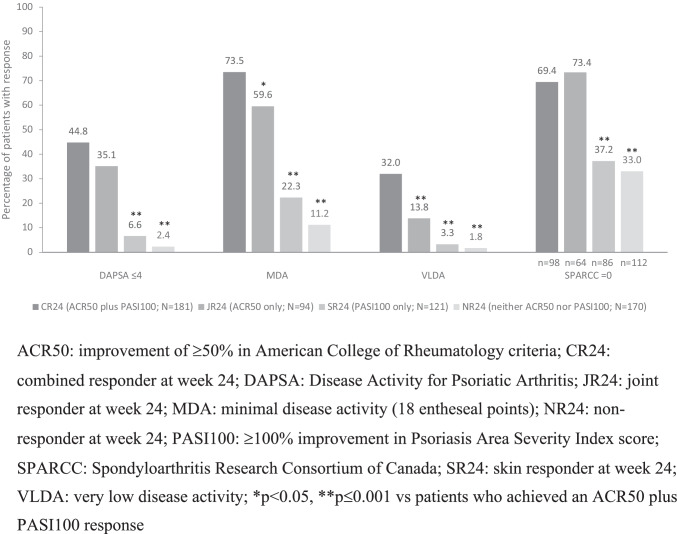
Rates of response for musculoskeletal endpoints at week 24 in each response group, irrespective of treatment

**Fig. 3:**
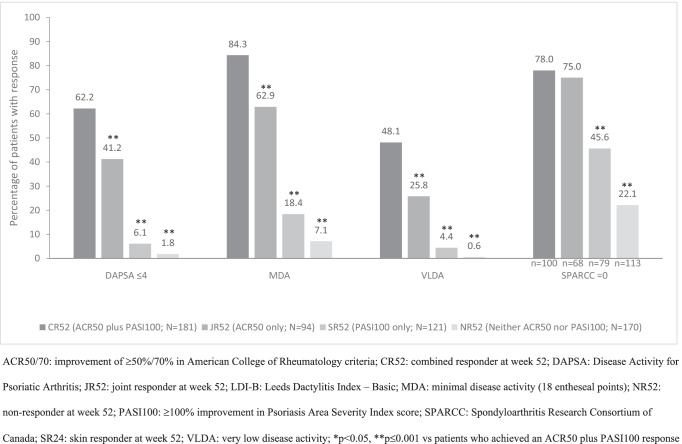
Rates of response for musculoskeletal and treat-to-target endpoints at week 52 in each response group, irrespective of treatment

Compared with patients in the other week-24 or week-52 response groups, patients in CR24 or CR52, respectively, achieved significantly (*p* < 0.05) higher rates of the musculoskeletal-related outcomes MDA and VLDA at weeks 24 and 52, respectively (Table 2, Figs. 2 and 3). DAPSA (≤ 14 or ≤ 4), enthesitis resolution, and HAQ-DI response rates were also numerically higher at weeks 24 and 52 in CR24 and CR52, respectively, than in the other corresponding response groups, being consistently significantly higher (*p* ≤ 0.001) than in SR24 and SR52, respectively, and NR24 and NR52, respectively. A similar pattern of relative differences between CR24 and JR24, and SR24 and NR24, and between CR52 and JR52, and SR52 and NR52 was seen for changes from baseline in enthesitis, HAQ-DI, and general HRQoL measured by SF-36 PCS and MCS scores at weeks 24 and 52, respectively (Table 2).

#### Musculoskeletal outcomes in patients achieving an ACR50 response only (JR24 and JR52)

Patients in JR24 or JR52, by definition, achieved both ACR20 and ACR50 responses at weeks 24 and 52, respectively. In addition, despite the numerically higher disease activity observed at baseline in these response subgroups, 48.9% and 60.8% of these response groups, respectively, also achieved ACR70 at weeks 24 and 52, respectively. Response rates for these outcomes, and for MDA, VLDA, DAPSA, enthesitis, and HAQ-DI, at week 24 were numerically higher in JR24 than in SR24 and NR24; similarly, corresponding response rates at week 52 were numerically higher in JR52 than in SR52 and NR52 (Table 2, Figs. 2 and 3).

#### Musculoskeletal outcomes in patients achieving a PASI100 response only (SR24 and SR52)

Musculoskeletal outcomes in patients achieving a PASI100 response only (SR24 and SR52) are summarised in the Online Resource and shown in Table 2 and Figs. 2 and 3.

#### Non-musculoskeletal outcomes

Considering non-musculoskeletal outcomes, patients in CR24 or CR52, by definition, achieved all PASI responses at weeks 24 and 52, respectively. The highest rates of complete nail resolution at weeks 24 and 52 were reported for these response groups, respectively (NAPSI = 0: 62.8% and 80.2%, respectively); a statistically significant difference was observed only in comparison with NR24 and NR52, respectively (Table 2). Patients in JR24 and JR52, although by definition not achieving a PASI100 response and having the numerically highest baseline PASI scores, achieved high rates of PASI75 response (60.6% at week 24 and 70.1% at week 52, respectively), and more than one-third of these patients also achieved a PASI90 response at week 24 or 52, respectively (Table 2). Many patients in JR24 or JR52 achieved complete resolution of nail symptoms at weeks 24 (50.0%) and 52 (67.6%), respectively. The highest rates of DLQI 0,1 were seen in groups with a PASI100 response (with or without an ACR50 response: CR24, SR24, CR52, and SR52) at both 24 and 52 weeks, although 47.9% of patients in JR24 and 47.4% of patients in JR52 achieved DLQI 0,1 at 24 and 52 weeks, respectively.

#### Comparison between week-24 and week-52 responses

**Fig. 4:**
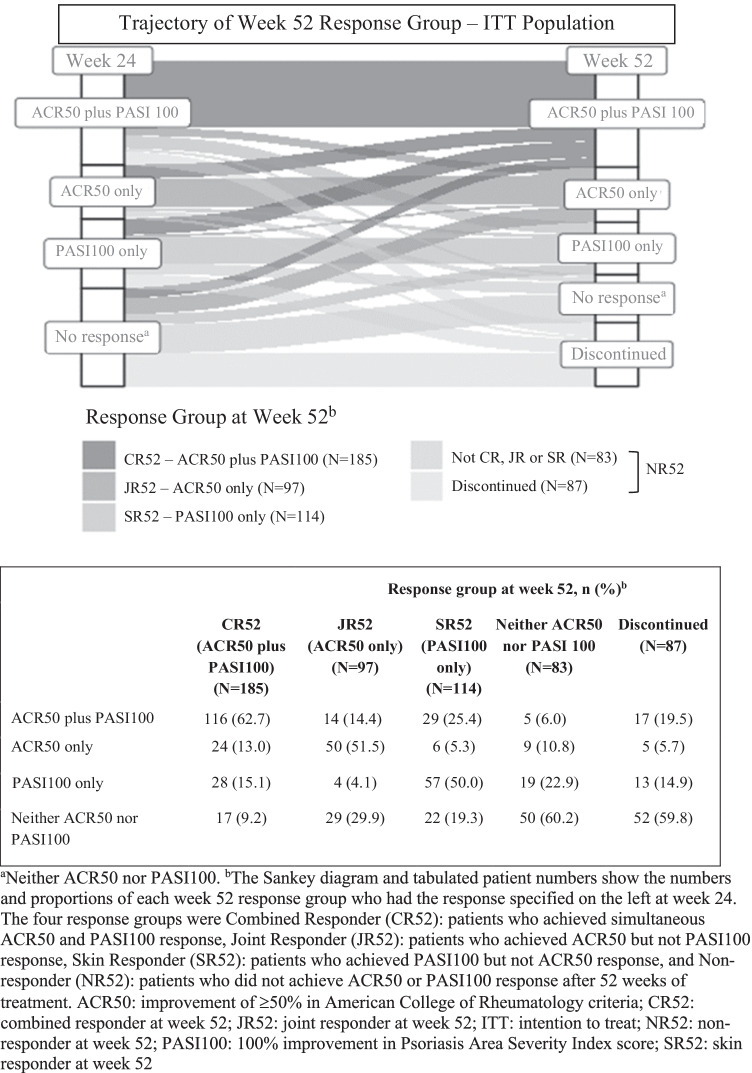
Sankey diagram showing trajectory of response based on response group at week 52

A majority (62.7%) of patients in CR52 had already achieved a simultaneous ACR50 and PASI100 response at week 24 (the primary endpoint); other patients in this group had achieved an ACR50 response only (13.0%) or a PASI100 response only (15.1%) at week 24 (Fig. 4). About half (51.5%) of the patients in JR52 had already achieved an ACR50 response only at week 24. The remaining patients in this latter group had neither an ACR50 nor a PASI100 response (29.9%), a PASI100 response only (4.1%) or a simultaneous ACR50 and PASI100 response (14.4%) at week 24.

Outcomes for each response group at week 24 and week 52 and the trajectory of response based on response group at week 52 by treatment group (ixekizumab versus adalimumab) are described in the Online Resource (Fig. S1 and Tables S2 and S3).

## Discussion

To optimise the benefits of treatment and improve the HRQoL of patients with PsA, all symptoms need to be considered [5]. Indeed, GRAPPA and EULAR recommendations indicate that the goal of treatment for patients with PsA is achievement of the lowest possible level of disease activity in all affected domains of the disease [2, 4]. Therefore, a number of unidimensional and multidimensional tools and treatment targets have been developed and validated to assess the musculoskeletal (articular and extra-articular) or non-musculoskeletal manifestations of PsA and the impact of disease on HRQoL. It would be useful if some of these measures, which encompass a variety of symptoms, could be combined to reflect treatment responses across a range of PsA manifestations.

The primary endpoint of the SPIRIT-H2H trial was a combination of both validated joint and skin measures [6]. ACR50 is a relatively stringent endpoint for musculoskeletal disease, and PASI100 is a very stringent endpoint for skin disease. The current analysis found that the simultaneous achievement of an ACR50 and PASI100 response, regardless of active treatment, reflected high response rates for additional musculoskeletal and non-musculoskeletal endpoints evaluated in SPIRIT-H2H and commonly used to assess treatments for PsA. In particular, patients in CR24 or CR52, who achieved simultaneous ACR50 and PASI100 at week 24 or 52, respectively, also achieved consistently high response rates for ACR70, MDA, VLDA, and DAPSA remission, and resolution of enthesitis, dactylitis, nail psoriasis, and impairment of physical function (HAQ-DI ≤ 0.5), thereby providing value to both patients and treating clinicians. Compared with the other corresponding response groups, including JR24 and JR52 (ACR50 response only), patients in CR24 or CR52 consistently achieved the highest response rates for specific musculoskeletal endpoints, non-musculoskeletal endpoints, and composite endpoints, and in many instances, the differences were statistically significant. These results indicate optimal control of PsA domains in the majority of patients who simultaneously achieved an ACR50 and PASI100 response, particularly at week 52. With regard to ACR 50 response at week 24, more male than female patients appeared to achieve this response. However, further analyses are warranted to determine a possible gender difference on disease presentation or severity, or on response to treatment, as has been reported previously [10–12].

Although HAQ-DI and SF-36 HRQoL endpoints improved to a greater extent in patients in JR24 or JR52 than in those in SR24 or SR52, respectively, the improvements were generally highest in CR24 and CR52, respectively, confirming the value of the combined endpoint. These SF-36 findings are possibly explained by improvements in joint function having a large impact on the PCS component of the SF-36, although improvement in the MCS was also greatest in patients with an ACR50 response, with or without a PASI100 response (CR24, JR24, CR52, and JR52). In contrast and as expected [13], the highest response rates for DLQI 0,1 were reported in patients who achieved a PASI100 response, irrespective of an ACR50 response (CR24, SR24, CR52, and SR52). However, as for the musculoskeletal endpoints, a DLQI 0,1 response was most likely to be observed in patients in CR24 or CR52.

We found that more than half of patients in SR24 or SR52 and about one-third of patients in NR24 or NR52 also achieved an ACR20 response at weeks 24 and 52, respectively. However, response rates for other musculoskeletal and composite endpoints at these times in these response groups were generally low.

EULAR recommends that consideration should be given to each musculoskeletal and non-musculoskeletal manifestation when managing patients with PsA, and that treatment decisions be made accordingly [2]. GRAPPA and the Outcome Measures in Rheumatology (OMERACT) association recommend that the core PsA domains to be assessed in patients with PsA are joint inflammation and damage, enthesitis, dactylitis, skin and nail disease, spondylitis, function, and HRQoL [13]. The combined endpoint evaluated in SPIRIT-H2H considered two of these domains: joint and skin activity. However, the current analysis shows that patients in CR24 or CR52 also had high response rates for endpoints relating to enthesitis, dactylitis and nail psoriasis resolution, functioning, and HRQoL, all core domains identified by GRAPPA-OMERACT, as well as the composite endpoints MDA, VLDA, and DAPSA remission.

Patients in CR52, JR52, or SR52 achieved response rates for other endpoints at week 52 that were generally similar to or higher than those achieved at week 24, indicating that the combined endpoint does not lose its association with other PsA endpoints over time. Results also indicate that continued treatment, even when a response is not achieved after 24 weeks of treatment, can be beneficial for many patients.

The current analysis was limited in that it was post hoc. In addition, as only small proportions of patients had dactylitis at baseline, LDI-B response rates should be interpreted with caution. As radiographic progression was not assessed in the SPIRIT-H2H trial, it was not possible to determine potential correlations between simultaneous achievement of ACR50 and PASI100 and inhibition of structural damage.

In conclusion, this post hoc analysis of data from the SPIRIT-H2H study showed that the primary endpoint of simultaneous achievement of an ACR50 and PASI100 response at week 24, irrespective of active treatment arm, signalled additional benefits at week 24 across a wide range of efficacy endpoints, including HRQoL, that are important for patients with PsA. Similar findings were seen in week-52 analyses. Patients meeting this primary endpoint are more likely to achieve a disease state of remission, which is the stated aim of PsA treatment according to GRAPPA and EULAR recommendations.

## Supplementary Information

Below is the link to the electronic supplementary material.

Supplementary file1 (PDF 284 KB)

## Data Availability

All data generated or analyzed during this study are included in this published article (and its supplementary information files).
