# Supplementary material for: Measuring treatment effect on psoriatic arthritis-related domains: insights from the SPIRIT-H2H study at weeks 24 and 52
Source: Clin Rheumatol. 2021 Sep 13;40(12):4943–54. doi: 10.1007/s10067-021-05891-5 (PMC8599337; doi:10.1007/s10067-021-05891-5)
Supplement: Supplementary file 1 — Supplementary file1 (PDF 284 KB) [file 10067_2021_5891_MOESM1_ESM.pdf]

Supplementary Material

Measuring treatment effect on psoriatic arthritis-related domains: insights from the SPIRIT-H2H study at weeks 24 and 52

Frank Behrens,[1] Soyi Liu Leage,[2] Christophe Sapin,[2] Celine El Baou,[2] Inmaculada De La Torre,[2] Gabriella Meszaros,[2] Georg Schett,[3,4] Bernard Combe,[5] Filip van den Bosch,[6] Laure Gossec[7,8]

Correspondence: PD Dr. med. Frank Behrens
Goethe University and Fraunhofer ITMP and CIMD, Frankfurt, Germany
Email: frank.behrens@ime.fraunhofer.de

Supplementary text

Additional results: definition of analysis groups and musculoskeletal outcomes in patients achieving a PASI100 response only and comparison between week 24 and week 52 responses.

Patients were classified into four independent analysis groups based on achievement of study endpoints, including a simultaneous 50% improvement in American College of Rheumatology criteria (ACR50) and 100% improvement in Psoriasis Area Severity Index (PASI100) response (the primary endpoint), using the overall SPIRIT-H2H intention-to-treat population, irrespective of treatment group. The four response groups were combined responder (CR24; patients who achieved simultaneous ACR50 and PASI100 response), joint responder (JR24; patients who achieved ACR50 but not PASI100 response), skin responder (SR24; patients who achieved PASI100 but not ACR50 response) and non-responder (NR24; patients who did not achieve ACR50 or PASI100 response after 24 weeks of treatment). Patients were also reallocated into the four groups based on responses after 52 weeks of treatment (CR52, JR52, SR52 and NR52, respectively). Patients who withdrew before week 24 or 52 were allocated to the groups who did not achieve an ACR50 or PASI100 response, based on non-responder imputation principles.

Musculoskeletal outcomes in patients achieving a PASI100 response only (SR24 and SR52)

Patients in SR24 or SR52, by definition, did not achieve an ACR50 response or greater. However, more than 50% of SR24 and SR52 achieved an ACR20 response at weeks 24 and 52, respectively. By contrast, response rates for achievement of minimal disease activity, very low disease activity, Disease Activity in Psoriatic Arthritis outcomes and Health Assessment Questionnaire–Disability Index improvements at weeks 24 and 52 were consistently low in these groups. Details of these findings are summarised in manuscript Table 2 and Figs. 2 and 3.

Comparison between week-24 and week-52 responses

For the response groups that included patients who achieved an ACR50 and/or PASI100 response (all groups except NR52 vs NR24), response rates for the majority of musculoskeletal and non-musculoskeletal outcomes were similar or numerically higher in the week-52 group than in the corresponding week-24 group (manuscript Table 2, Figs. 2 and 3). For all response groups, least squares mean improvement from baseline in musculoskeletal outcome scores was generally similar at week 24 and week 52 in the corresponding response groups (CR24 vs CR52, JR24 vs JR52, SR24 vs SR52 and NR24 vs NR52), but nail improvement was numerically greater at week 52 than week 24 in all the groups with an ACR50 and/or PASI100 response (CR52 vs CR24, JR52 vs JR24 and SR52 vs SR24; Table 2).

Measuring treatment effect on psoriatic arthritis-related domains: insights from the SPIRIT-H2H study at weeks 24 and 52

Frank Behrens,[1] Soyi Liu Leage,[2] Christophe Sapin,[2] Celine El Baou,[2] Inmaculada De La Torre,[2] Gabriella Meszaros,[2] Georg Schett,[3,4] Bernard Combe,[5] Filip van den Bosch,[6] Laure Gossec[7,8]

Correspondence: PD Dr. med. Frank Behrens
Goethe University and Fraunhofer ITMP and CIMD, Frankfurt, Germany
Email: frank.behrens@ime.fraunhofer.de

Fig. S1 Sankey diagram showing trajectory of response based on response group at week 52, by treatment. A simultaneous ACR50 and PASI100 response was achieved by 39.6% of ixekizumab- and 26.1% of adalimumab-treated patients at week 52.

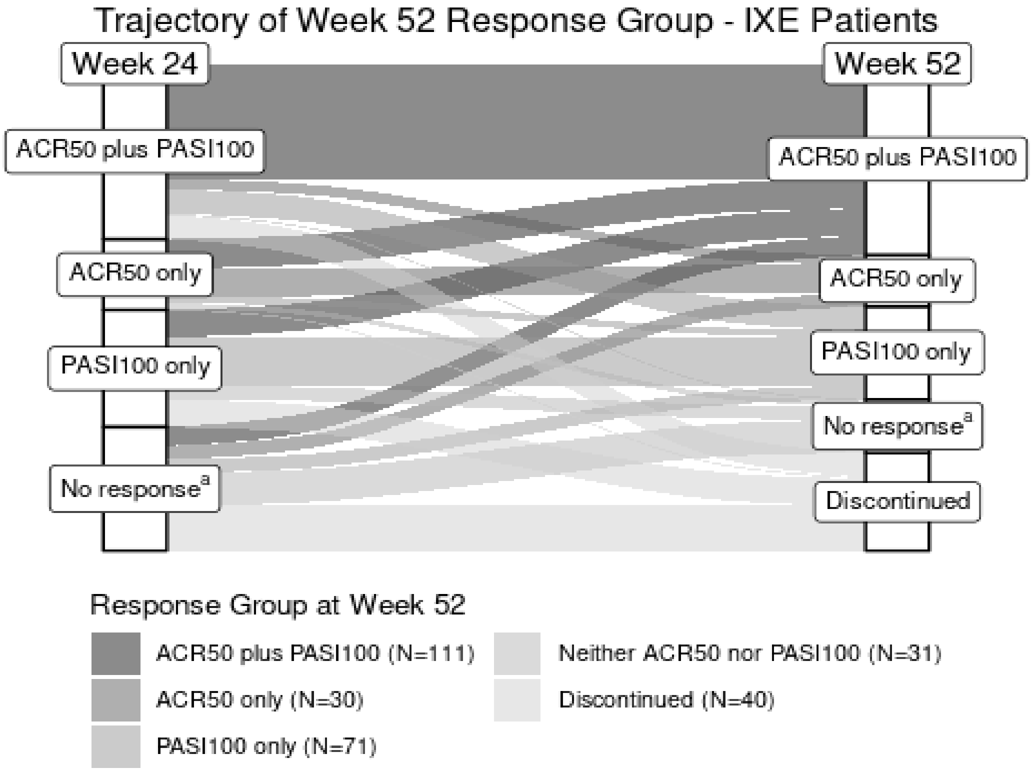

**Ixekizumab (N=283)**	**Response group at week 52, n (%)**				
	**ACR50 plus PASI100 (N=111)**	**ACR50 only (N=30)**	**PASI100 only (N=71)**	**Neither ACR50 nor PASI 100 (N=31)**	**Discontinued (N=40)**

Response group at week 24					
ACR50 plus PASI100	67 (60.4)	6 (20.0)	20 (28.2)	1 (3.2)	8 (20.0)
ACR50 only	17 (15.3)	16 (53.3)	4 (5.6)	3 (9.7)	1 (2.5)
PASI100 only	16 (14.4)	0	37 (52.1)	8 (25.8)	7 (17.5)
Neither ACR50 nor PASI100	11 (9.9)	8 (26.7)	10 (14.1)	19 (61.3)	24 (60.0)

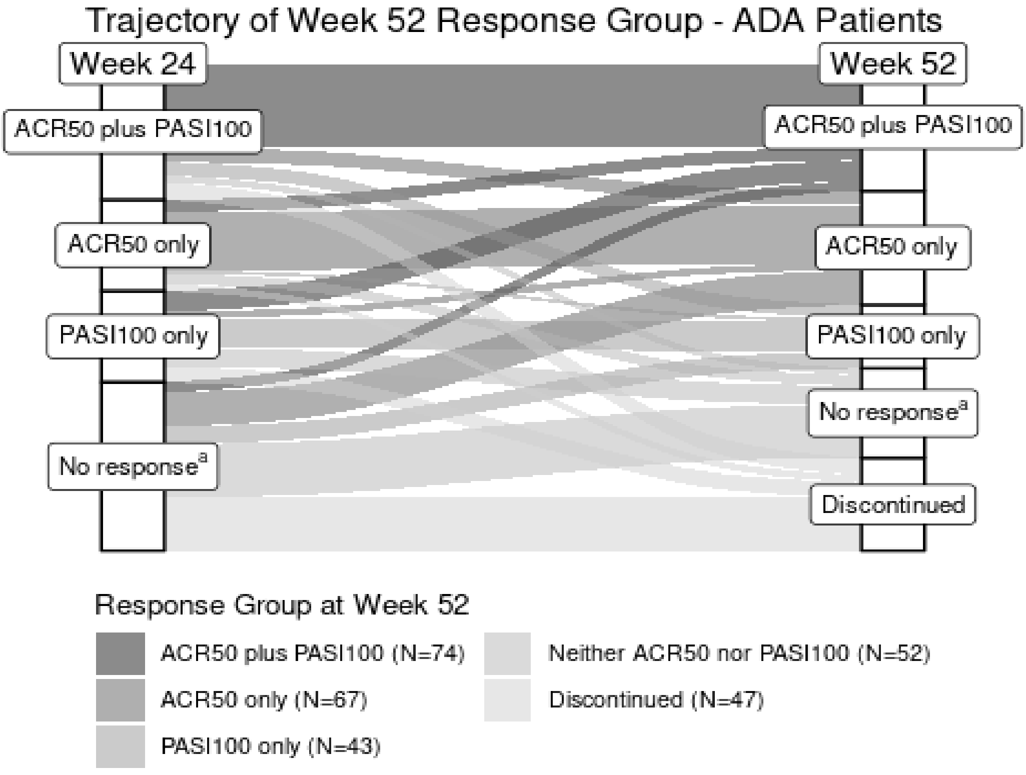

**Adalimumab (N=283)**	**Response group at week 52, n (%)**				
	**ACR50 plus PASI100 (N=74)**	**ACR50 only (N=67)**	**PASI100 only (N=43)**	**Neither ACR50 nor PASI 100 (N=52)**	**Discontinued (N=47)**
Response group at week 24					

ACR50 plus PASI100	49 (66.2)	8 (11.9)	9 (20.9)	4 (7.7)	9 (19.1)
ACR50 only	7 (9.5)	34 (50.7)	2 (4.7)	6 (11.5)	4 (8.5)
PASI100 only	12 (16.2)	4 (6.0)	20 (46.5)	11 (21.2)	6 (12.8)
Neither ACR50 nor PASI100	6 (8.1)	21 (31.3)	12 (27.9)	31 (59.6)	28 (59.6)

[a]Neither ACR50 nor PASI100. [b]The Sankey diagram and tabulated patient numbers show the numbers and proportions of each week 52 response group who had the response specified on the left at week 24.

ACR50: improvement of ≥50% in American College of Rheumatology criteria; ADA: adalimumab; IXE: ixekizumab; PASI100: 100% improvement in Psoriasis Area Severity Index score

Measuring treatment effect on psoriatic arthritis-related domains: insights from the SPIRIT-H2H study at weeks 24 and 52

Frank Behrens,[1] Soyi Liu Leage,[2] Christophe Sapin,[2] Celine El Baou,[2] Inmaculada De La Torre,[2] Gabriella Meszaros,[2] Georg Schett,[3,4] Bernard Combe,[5] Filip van den Bosch,[6] Laure Gossec[7,8]

Correspondence: PD Dr. med. Frank Behrens

Goethe University and Fraunhofer ITMP and CIMD, Frankfurt, Germany

Email: frank.behrens@ime.fraunhofer.de

Table S1 Baseline characteristics of week-52 response groups[a]

	CR52 (ACR50 plus PASI100) (N=185)	JR52 (ACR50 only) (N=97)	SR52 (PASI100 only) (N=114)	NR52 (neither ACR50 nor PASI 100) (N=170)
Age (years)	46.3 ± 11.9	47.6 ± 11.8	49.0 ± 12.7	49.1 ± 12.2
Sex, male	116 (62.7)	62 (63.9)	57 (50.0)	77 (45.3)
BMI (kg/m^2)	28.6 ± 5.6	30.5 ± 8.3	29.4 ± 6.9	31.1 ± 9.2
Duration of symptoms (years)				

since Ps diagnosis	14.3 ± 11.9	15.4 ± 12.3	14.4 ± 12.7	17.2 ± 14.1
since PsA diagnosis	6.2 ± 6.5	6.2 ± 5.9	5.3 ± 5.5	6.9 ± 8.5
csDMARD use	135 (73.0)	66 (68.0)	76 (66.7)	115 (67.6)
Methotrexate use	116 (62.7)	59 (60.8)	65 (57.0)	96 (56.5)
Glucocorticoid use	34 (18.4)	17 (17.5)	25 (21.9)	35 (20.6)
Tender Joint Count	19.1 ± 13.5	21.4 ± 15.2	19.6 ± 14.6	21.1 ± 13.9
Swollen Joint Count	10.6 ± 8.3	11.7 ± 8.8	9.8 ± 6.4	9.8 ± 7.3
Physician's Disease Activity VAS (mm)	59.4 ± 18.5	62.5 ± 17.4	57.0 ± 17.1	58.3 ± 17.7
Patient's Disease Activity VAS (mm)	62.7 ± 19.5	67.8 ± 19.4	59.8 ± 22.1	65.3 ± 21.0
Patient's Pain VAS (mm)	60.7 ± 20.8	64.4 ± 20.0	57.0 ± 23.3	62.1 ± 21.8
HAQ-DI score	1.2 ± 0.6	1.4 ± 0.7	1.1 ± 0.7	1.3 ± 0.7

CRP level (mg/L)	12.5 ± 21.3	10.0 ± 15.9	6.8 ± 9.7	10.0 ± 14.6
CRP level >6 mg/L	81 (44.0)	41 (44.1)	35 (31.8)	61 (37.0)
DAPSA score	43.3 ± 21.6	47.9 ± 24.7	42.0 ± 21.4	44.8 ± 21.5
DAS28-CRP score	4.8 ± 1.1	5.0 ± 1.0	4.6 ± 1.0	4.9 ± 1.0
LEI >0	90 (48.6)	56 (57.7)	68 (59.6)	92 (54.4)
LEI score[b]	2.5 ± 1.6	2.6 ± 1.4	2.4 ± 1.4	2.9 ± 1.3
SPARCC >0	100 (54.1)	68 (70.1)	79 (69.3)	113 (66.9)
SPARCC score[b]	5.1 ± 3.9	5.2 ± 3.5	4.9 ± 3.3	5.7 ± 3.7
LDI-B >0	34 (18.4)	19 (19.6)	16 (14.0)	31 (18.3)
LDI-B score[b]	29.2 ± 22.1	87.6 ± 198.3	48.5 ± 42.0	47.9 ± 87.0
mCPDAI score	6.0 ± 2.0	6.6 ± 2.2	5.9 ± 1.9	6.2 ± 2.0

PASI score	7.6 ± 8.2	10.5 ± 9.1	6.2 ± 6.7	7.6 ± 7.7
sPGA score	2.6 ± 0.9	3.0 ± 0.8	2.5 ± 0.8	2.7 ± 0.9
BSA score (% affected)	13.2 ± 16.6	20.3 ± 20.7	11.2 ± 14.8	12.6 ± 15.9
NAPSI >0	121 (65.4)	68 (70.8)	71 (62.3)	108 (63.5)
NAPSI score[b]	19.4 ± 16.9	20.5 ± 18.0	20.3 ± 18.4	18.1 ± 17.1
DLQI score	9.8 ± 7.9	12.3 ± 7.7	8.2 ± 7.0	9.4 ± 7.2
SF-36 PCS	37.6 ± 8.2	35.6 ± 8.1	39.3 ± 8.7	35.6 ± 8.6
SF-36 MCS	45.0 ± 10.8	41.5 ± 11.4	43.6 ± 10.9	45.8 ± 11.8

Results are expressed as mean ± SD or n (%); percentages are calculated based on numbers of patients providing data.

[a]The four response groups were combined responder (CR52; patients who achieved simultaneous ACR50 and PASI100 response), joint responder (JR52; patients who achieved ACR50 but not PASI100 response), skin responder (SR52; patients who achieved PASI100 but not ACR50 response) and non-responder (NR52; patients who did not achieve ACR50 or PASI100 response) after 52 weeks of treatment.

[b]Mean ± SD score in patients with baseline score >0 (see row above for number of patients evaluated).

ACR50: ≥50% improvement in American College of Rheumatology criteria; BMI: body mass index; BSA: body surface area; CR52: combined responder at week 52; CRP: C-reactive protein; csDMARD: conventional synthetic disease-modifying antirheumatic drug; DAPSA: Disease Activity for Psoriatic Arthritis; DAS28-CRP: Disease Activity Score with CRP; DLQI: Dermatology Life Quality Index; HAQ-DI: Health Assessment Questionnaire–Disability Index; JR52: joint responder at week 52; LDI-B: Leeds Dactylitis Index – Basic; LEI: Leeds Enthesitis Index; mCPDAI: modified Composite Psoriatic Disease Activity Index (without spinal disease assessment); MCS: mental component score; NAPSI: Nail Psoriasis Severity Index; NR52: non-responder at week 52; PASI: Psoriasis Area Severity Index; PASI100: 100% improvement in PASI; PCS: physical component score; Ps: psoriasis: PsA: psoriatic arthritis; SD: standard deviation; SF-36: 36-Item Short Form Survey; SPARCC: Spondyloarthritis Research Consortium of Canada; sPGA: static Physician's Global Assessment of Psoriasis; SR52: skin responder at week 52; VAS: visual analogue scale.

Measuring treatment effect on psoriatic arthritis-related domains: insights from the SPIRIT-H2H study at weeks 24 and 52

Frank Behrens,[1] Soyi Liu Leage,[2] Christophe Sapin,[2] Celine El Baou,[2] Inmaculada De La Torre,[2] Gabriella Meszaros,[2] Georg Schett,[3,4] Bernard Combe,[5] Filip van den Bosch,[6] Laure Gossec[7,8]

Correspondence: PD Dr. med. Frank Behrens

Goethe University and Fraunhofer ITMP and CIMD, Frankfurt, Germany

Email: frank.behrens@ime.fraunhofer.de

Table S2 Efficacy and health-related quality-of-life outcomes at week 24 in each week 24 response group, by treatment group

Week 24 response group								
	**Ixekizumab (% of patients)**				**Adalimumab (% of patients)**			
	**ACR50 plus PASI100 (N=102)**	**ACR50 only (N=41)**	**PASI100 only (N=68)**	**None (N=72)**	**ACR50 plus PASI100 (N=79)**	**ACR50 only (N=53)**	**PASI100 only (N=53)**	**None (N=98)**
ACR50/PASI100	100	0**	0**	0**	100	0**	0**	0**

ACR20	100	100	48.5**	26.4**	100	100	60.4**	40.8**
ACR50	100	100	0**	0**	100	100	0**	0**
ACR70	68.6	48.8*	0**	0**	59.5	49.1	0**	0**
MDA	81.4	70.7	20.6**	12.5**	63.3	50.9	24.5**	10.2**
VLDA	34.3	22.0	5.9**	1.4**	29.1	7.5*	0**	2.0**
DAPSA ≤4	49.0	41.5	8.8**	2.8**	39.2	30.2	3.8**	2.0**
DAPSA ≤14	93.1	87.8	39.7**	22.2**	91.1	77.4*	47.2**	33.7**
LEI =0[a]	n=54<br>75.9	n=21<br>81.0	n=44<br>50.0*	n=40<br>37.5**	n=33<br>78.8	n=33<br>72.7	n=27<br>37.0**	n=54<br>38.9**
SPARCC =0[a]	n=63<br>73.0	n=26<br>76.9	n=54<br>46.3*	n=46<br>34.8**	n=35<br>62.9	n=38<br>71.1	n=32<br>21.9**	n=66<br>31.8*
LDI-B =0[a]	n=18<br>100.0	n=5<br>80.0	n=9<br>88.9	n=10<br>70.0*	n=16<br>100	n=15<br>100	n=12<br>100	n=15<br>73.3*
HAQ-DI ≤0.5	80.4	70.7	26.5**	20.8**	69.6	60.4	34.0**	28.6**

HAQ-DI MCID improvement ≥0.35	84.3	82.9	45.6**	23.6**	79.7	84.9	35.8**	39.8**
PASI75	100	63.4**	100	43.1**	100	58.5**	100	32.7**
PASI90	100	43.9**	100	20.8**	100	30.2**	100	10.2**
PASI100	100	0**	100	0**	100	0**	100	0**
NAPSI =0[a]	n=67<br>70.1	n=34<br>55.9	n=50<br>60.0	n=40<br>37.5**	n=46<br>52.2	n=36<br>44.4	n=33<br>57.6	n=62<br>46.8
DLQI 0,1	81.4	53.7**	67.6*	31.9**	72.2	43.4**	73.6	28.6**

ACR20/50/70: improvement of ≥20%/50%/70% in American College of Rheumatology criteria; CR24/52: combined responder at week 24/52; DAPSA: Disease Activity for Psoriatic Arthritis; DLQI: Dermatology Life Quality Index; HAQ-DI: Health Assessment Questionnaire–Disability Index; JR24/52: joint responder at week 24/52; LDI-B: Leeds Dactylitis Index – Basic; LEI: Leeds Enthesitis Index; MCID: minimal clinically important difference; MDA, minimal disease activity; NAPSI: Nail Psoriasis Severity Index; NR24/52: non-responder at week 24/52; PASI75/90/100: ≥75%/90%/100% improvement in Psoriasis Area Severity Index score; SPARCC: Spondyloarthritis Research Consortium of Canada; SR24/52: skin responder at week 24/52; VLDA, very low disease activity; *p<0.05, **p≤0.001 vs ACR50 plus PASI100 response group

[a]Percentage of patients achieving outcome/mean ± SD improvement in score in patients with baseline score>0.

Measuring treatment effect on psoriatic arthritis-related domains: insights from the SPIRIT-H2H study at weeks 24 and 52

Frank Behrens,[1] Soyi Liu Leage,[2] Christophe Sapin,[2] Celine El Baou,[2] Inmaculada De La Torre,[2] Gabriella Meszaros,[2] Georg Schett,[3,4] Bernard Combe,[5] Filip van den Bosch,[6] Laure Gossec[7,8]

Correspondence: PD Dr. med. Frank Behrens

Goethe University and Fraunhofer ITMP and CIMD, Frankfurt, Germany

Email: frank.behrens@ime.fraunhofer.de

Table S3 Efficacy and health-related quality-of-life outcomes at week 52 in each week 52 response group, by treatment group

	**Ixekizumab (% of patients)**				**Adalimumab (% of patients)**			
	**ACR50 plus PASI100 (N=111)**	**ACR50 only (N=30)**	**PASI100 only (N=71)**	**None (N=71)**	**ACR50 plus PASI100 (N=74)**	**ACR50 only (N=67)**	**PASI100 only (N=43)**	**None (N=99)**
ACR50/PASI100	100	0**	0**	0**	100	0**	0**	0**
ACR20	100	100	56.3**	22.5**	100	100	51.2**	32.3**
ACR50	100	100	0**	0**	100	100	0**	0**
ACR70	76.6	50.0*	0**	0**	71.6	65.7	0**	0**
MDA	87.4	63.3*	19.7**	5.6**	79.7	62.7*	16.3**	8.1**

VLDA	46.8	30.0	7.0**	0**	50.0	23.9*	0**	1.0**
DAPSA ≤4	60.4	40.0	8.5**	0**	64.9	41.8*	2.3**	3.0**
DAPSA ≤14	92.8	90.0	43.7**	18.3**	97.3	89.6	32.6**	20.2**
LEI =0[a]	n=58<br>82.8	n=18<br>83.3	n=44<br>50.0**	n=39<br>33.3**	n=32<br>87.5	n=38<br>84.2	n=24<br>41.7**	n=53<br>26.4**
SPARCC =0[a]	n=66<br>80.3	n=23<br>73.9	n=49<br>51.0**	n=51<br>23.5**	n=34<br>73.5	n=45<br>75.6	n=30<br>36.7*	n=62<br>21.0**
LDI-B =0[a]	n=15<br>100	n=5<br>100	n=11<br>90.9	n=11<br>45.5*	n=19<br>100	n=14<br>100	n=5<br>60.0*	n=20<br>55.0**
HAQ-DI ≤0.5	78.4	56.7*	35.2**	15.5**	78.4	64.2	32.6**	14.1**
HAQ-DI MCID improvement ≥0.35	82.9	83.3	53.5**	18.3**	83.8	80.6	39.5**	31.3**
PASI75	100	70.0**	100	26.8**	100	70.1**	100	30.3**
PASI90	100	40.0**	100	16.9**	100	31.3**	100	15.2**
PASI100	100	0**	100	0**	100	0**	100	0**
NAPSI =0[a]	n=78<br>79.5	n=22<br>59.1	n=45<br>82.2	n=46<br>37.0**	n=43<br>81.4	n=46<br>71.7	n=26<br>57.7	n=62<br>33.9**
DLQI 0,1	82.0	53.3*	62.0*	22.5**	82.4	44.8**	67.4	18.2**

ACR20/50/70: improvement of ≥20%/50%/70% in American College of Rheumatology criteria; CR24/52: combined responder at week 24/52; DAPSA: Disease Activity for Psoriatic Arthritis; DLQI: Dermatology Life Quality Index; HAQ-DI: Health Assessment Questionnaire–Disability Index; JR24/52: joint responder at week 24/52; LDI-B: Leeds Dactylitis Index – Basic; LEI: Leeds Enthesitis Index; MCID: minimal clinically important difference; MDA, minimal disease activity; NAPSI: Nail Psoriasis Severity Index; NR24/52: non-responder at week 24/52; PASI75/90/100: ≥75%/90%/100% improvement in Psoriasis Area Severity Index score; SPARCC: Spondyloarthritis Research Consortium of Canada; SR24/52: skin responder at week 24/52; VLDA, very low disease activity; *p<0.05, **p≤0.001 vs ACR50 plus PASI100 response group

[a]Percentage of patients achieving outcome/mean ± SD improvement in score in patients with baseline score>0.
